# Performance Analysis of Autonomous Microgrid Subsequent to Symmetrical and Unsymmetrical Fault Triggered Condition

**DOI:** 10.1155/2014/715963

**Published:** 2014-08-04

**Authors:** Chitra Natesan, Senthil Kumar Ajithan, Shobana Mani, Priyadharshini Palani, Prabaakaran Kandhasamy

**Affiliations:** ^1^Department of Electrical & Electronics Engineering, SKP Engineering College, Anna University & Asso., Tiruvannamalai 606611, India; ^2^Department of Electrical & Electronics Engineering, Velammal Engineering College, Chennai 600066, India; ^3^Department of Electrical & Electronics Engineering, Sri Rangapoopathi College of Engineering, Gingee 604151, India

## Abstract

Hi-tech scenario and the ecological compression are the key point to drive the intervention of the renewable in the distribution system. In the perspective of complex power system planners, the transient performance of the microgrid is the main concern. For that purpose, various fault cases are explored in order to examine the microgrid transient performance when subjected to accidental events. In this work, the microgrid is modelled with two distributed generations (DGs) tied with a converter separately. With this intention, droop control strategy is adopted for the microsources to examine the microgrid performance during the symmetrical and unsymmetrical fault events. The ability of the control strategy adopted in this work and its effectiveness are evaluated through Matlab/Simulink platform.

## 1. Introduction

A new concept of microgrid (MG) was introduced with the help of power electronic devices and renewable energy sources. DG is considered to be a supplementary source of power in addition to the centralized power generation. Microsources and distributed generation combined with controllers, loads, and storage devices in order to compose a microgrid structure. Usually the microgrid sources are diesel generators, fuel cells, solar cells, and wind turbine. Distributed generations are usually combined with power electronic devices to get contact with the microgrid. DG interfaced with inverter can reduce oscillation throughout its fast control action [1].

Grid-tied mode and autonomous mode are the functions of microgrid system on the basis of static transfer switch position [2]. In case of any interruption, the microgrid should work on island mode to meet the demand in load. Also it should maintain power quality accordingly [3].

In case of real time, DG will breakdown accidentally or sometimes intentionally. So it has to provide uninterrupted supply; the microgrid has to sense mode of operation and switch to island mode in order to meet voltage supply for sensitive devices. This scenario is tough in real time for the power companies to provide reliable power supply [4]. But, in current industrial plot, it must detach DGs for the safety purpose. It also retards from switching to island mode to maintain protection constraints in distribution system.

Various research papers are discussed under the concept of transient stability subsequent to fault condition [5–11].

The author in [5] explores a new perspective by introducing different control strategies such as master slave approach and droop control approach for the transient stability analysis in a microgrid subsequent to fault triggered incident. In this work, diesel based generators and inverter based DG are composed. For inverter based DG, two control schemes such as real and reactive power (*P* and *Q*) control and current control scheme are introduced. Further, the author concluded that the droop control approach is best suited for subsequent transient incidents and *P* and *Q* control scheme holds good for inverter based DG.

Since the mechanism of microgrid operation differs from the traditional power grid, it is necessary to opt for the separate transient performance analysis [7]. So a dynamic model is projected for a microgrid to promote the difference among the microgrid and traditional grid transient analysis. In this work, differential algebraic equation is composed in order to model the dynamic system.

A new dimension of approach was conferred [8] about the transient stability in the course of network disruption in a wind farm when it is connected to a complex power system. To enhance the transient stability performance, superconducting magnetic energy storage (SMES) based controlled adaptive artificial neural network (ANN) was adopted. The SMES function on the basis of three criterions such as (i) voltage source converter (VSC), (ii) sinusoidal pulse width modulation (PWM), and (iii) an ANN controlled DC-DC converter using insulated gate bipolar transistors (IGBTs). Further, the ANN is compared with proportional integral (PI) on the basis of SMES control which is optimized by response surface methodology and genetic algorithm (RSM-GA) in view of both symmetrical and asymmetrical faults. Hence, in conclusion, the transient performance is better while adapting ANN controller than PI controller.

Another interesting technique was categorized in [9]; the effectiveness of two different models of doubly fed induction generator (DFIG) wind turbine is compared, that is, DFIG with dynamic voltage restorer (DVR) and DFIG with crowbar protection under different fault conditions. In the model of DFIG with crowbar protection, the reactive power production was found to be more complicated, whereas the model of DFIG with DVR helps to compensate the fault voltage so that the DFIG wind turbine provides an uninterruptable power supply as per the load demand without the need of any additional protection.

Another novel approach attempted in [10] is the enhancement of microgrid performance during fault conditions with the battery energy storage system controller (BESS). But it has the restrictions for the sustainable operation; that is, the remnant energy of BESS must be zero.

The author in [11] introduced a dynamic model for the general microgrid under different conditions such as island operation, emergency control function, and service restoration in case of block-out. Therefore, the author defined a new control procedure for microgrid operation and management in LV distribution networks. In addition, controllable loads are also modelled, which are in desperate need of load shedding.

When *n* number of inverter based DGs are connected in parallel to a microgrid system, there may raise problem in standalone operation, that is, less inertia which leads to either supply or consumption of energy during transient period. To resolve this problem, the author in [6] theoretically studied the small signal stability through Eigen value analysis.

It is compulsory to evaluate transient instability in order to prevent transient overloads. Highly dangerous transient overloads take part even though microsources are interconnected with power electronic devices [12]. Microgrid works parallel to main grid unless there is a fault in the system. It is hard to protect system from fault condition in case of grid connected mode. So the microgrid will operate separately after the fault event [13].

**Figure 1:**
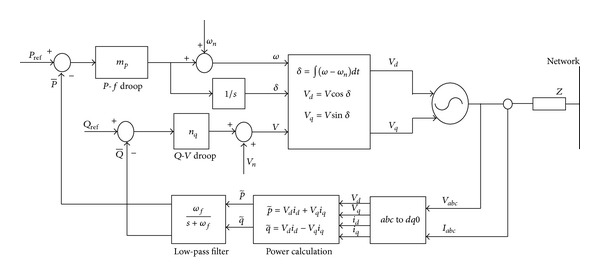
Droop control block diagram.

Concerning these common transient issues, a control strategy is developed to examine the performance of the microgrid. *P*-*F* and *Q*-*V* droop control are adopted to examine its impact during the transient stability period (Figure 1). Generally, the main use of the above method is to share appropriate power to DGs with the help of maintaining frequency and voltage [14, 15].

The impact of droop control technique and the stability of the DG based on inverter arrangement of microgrid consequent to fault-forced autonomous circumstances are examined in this work. Further, the microgrid stability performance is scrutinised with induction motor (IM) loads. MatLab/Simulink and its libraries (mainly the sim power systems toolbox) were employed in order to develop a simulation platform suitable for identifying MG control requirements and evaluating MG dynamic behaviour under several operating conditions.

## 2. Control Strategies

In microgrid, the system reliability and stability are achieved only by the voltage regulation when more microsources are interconnected. This voltage regulation damps the reactive power oscillations and voltage [16]. In a complex power system, when multiple DGs are attached to the microgrid, the power sharing among them is made properly with the help of a control strategy called droop control. Droop control also enables the system to disconnect smoothly and reconnect routinely to the complex power system [17].

The role of droop control in this work is that it controls the real power on the basis of frequency droop control and it controls the reactive power on the basis of voltage control [18, 19]. The voltage and frequency can be manipulated by regulating the real and reactive power of the system. This forms a droop control equation.

In a transmission line, the real and reactive power are designed as

(1) $$\begin{array}{c} P=\frac{V1V2}{X}\mathrm{Sin}\delta ,\\ Q=\frac{{V1}^{2}}{X}-\frac{V1V2}{X}\mathrm{Cos}\delta . \end{array}$$

In the abovementioned equation (1), resistance (*R*) is neglected for overhead transmission lines as it is much lower than inductance (*L*). Also the power angle *δ* is lesser. Therefore, sin *δ* = *δ* and cos *δ* = 1.

(2) $$\begin{array}{c} \delta =\frac{XP}{V1V2},\\ V1-V2≅\frac{XQ}{V1}. \end{array}$$

Hence, from the above equation (2), it is clear that the power angle *δ* can be controlled by regulating real power *P*. Also, the voltage *V*1 can be controlled through reactive power *Q*. Dynamically, the frequency control leads to regulating the power angle and this in turn controls the real power flow [20]. Finally, the frequency and voltage amplitude of the microgrid are manipulated by adjusting the real and reactive power autonomously. As a result, the frequency and voltage droop regulation can be determined as

(3) $$\begin{array}{c} f-f_{0}=K_{p}\left(P-P_{0}\right),\\ V-V_{0}=K_{q}\left(Q-Q_{0}\right). \end{array}$$

The relationship between real power, frequency, and reactive power, voltage, can be manipulated from (3):

(4) $$\begin{array}{c} f=f_{0}+K_{p}\left(P-P_{0}\right),\\ V=V_{0}+K_{q}\left(Q-Q_{0}\right), \end{array}$$

where *f*,  *V* = the frequency and voltage at a new operating point. *P*,  *Q* = active and reactive power at a new operating point. *f*_0_,  *V*_0_ = base frequency and voltage. *P*_0_,  *Q*_0_ = temporary set points for the real and reactive power. *K*_*p*_,  *K*_*q*_ = droop constant.

## 3. Stability Analysis

In recent researches, the stability issue in the microgrid seems to be gaining its attention. The power system engineers chiefly focus the restructuring in the power system particularly when exposed to a severe disturbance. Typically, the system designers target the aspect of reliable and stable power supply as per their load demands [21, 22]. Among various stability issues of microgrid, transient stability alone is considered in this work. In course of islanding process, the transient effects depend on the criterions such as (i) working conditions before islanding and (ii) the events which cause islanding. Even though it may be planned or unplanned islanding, the microgrid is expected to stay in working condition. Therefore, it is necessary to enhance the dynamic response of the microgrid [23].

### 3.1. Transient Stability

**Figure 2:**
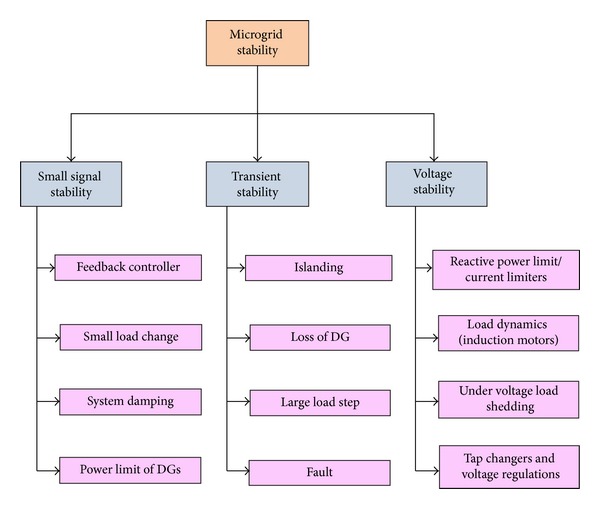
Different stability issues in microgrid.

**Figure 3:**
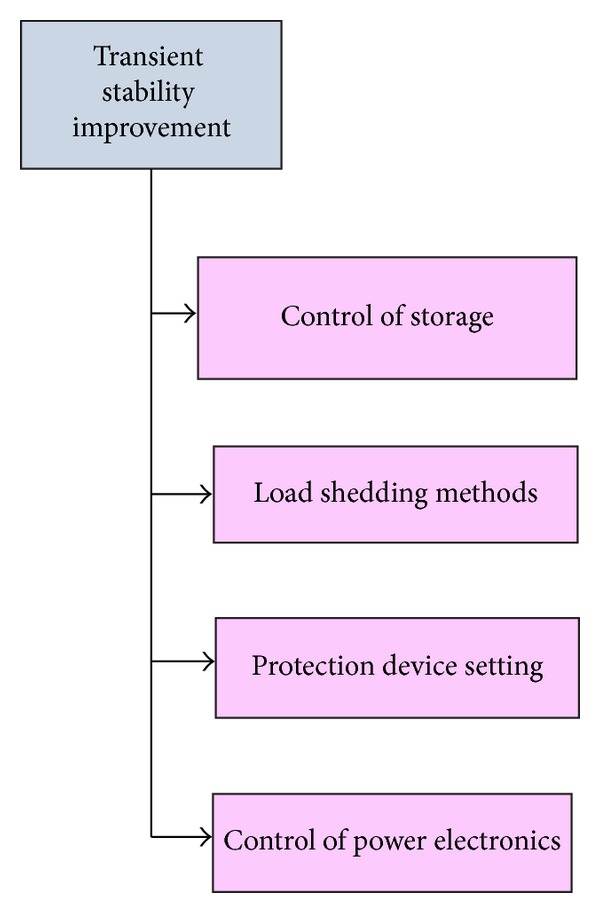
Different transient stability improvement methods.

Even at more disturbances, the microgrid can perform its action without any interventions. This function changes the microgrid mode from grid-tied mode to island mode. There is a possibility of occurrence of heavy disturbance in power system due to switching of heavy loads or transmission line fault. Hence, this disturbance will lead to synchronisation loss in the system. It is represented as transient stability [24]. In recent times, many system stability issues have been researched and some stability improvement methods have been developed which are shown in Figures 2 and 3. Control strategies, microsources, and type of microgrid are the main parameters of stability. All the microsources are connected to power electronic devices, that is, here voltage source inverter. So the control method of power electronic devices controls the stability [25, 26].

## 4. Simulation Model

**Figure 4:**
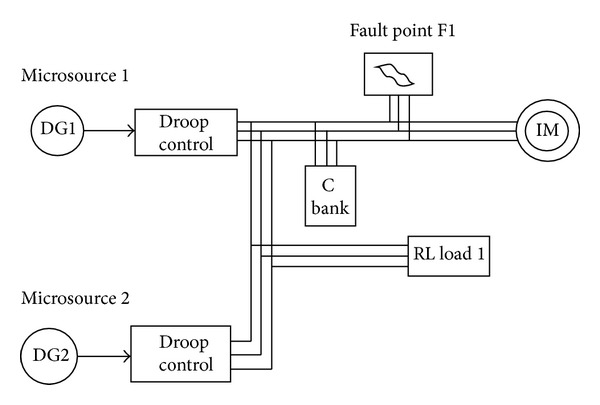
Single line layout of the utility and microgrid systems.

**Figure 5:**
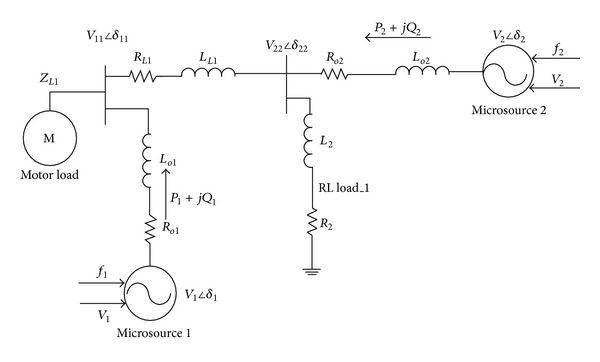
Equivalent model of the microgrid systems.

The microgrid is modelled with two microsources which have a DC source as input and the *P*-*F* and *Q*-*V* droop control have been employed. At the time of starting, RL load is connected to the system as shown in Figure 4 and its equivalent model is shown in Figure 5. In order to highlight the performance difference of microgrid when connected with normal load and nonlinear load, induction motor is connected at 0.75 sec and disconnected at 2.5 sec. As a next, study about the activity of microgrid with RLload during fault triggered incident is analyzed. To perform this activity, a LG fault was introduced for a period of 1 to 1.5 secs. This microgrid system has been simulated under the Matlab/Simulink software environs.

## 5. Simulation Result 

With the intention of authenticating the aforesaid control strategies, AC system was programmed to evaluate its performance when subjected to various faults. The symmetrical and unsymmetrical fault analysis was implemented in a Matlab/Simulink platform. The parameters such as real and reactive power, fault voltage, fault current, and the rotor speed waveforms are presented for all the faults in Figures 4 and 5.

**Figure 6:**
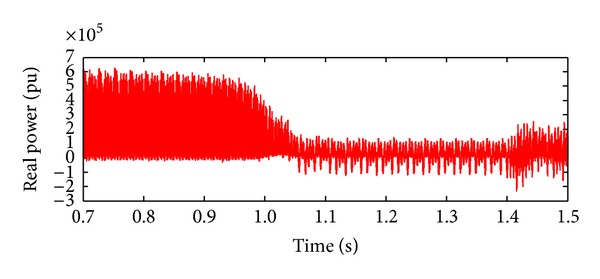
Real power during L-G fault.

**Figure 7:**
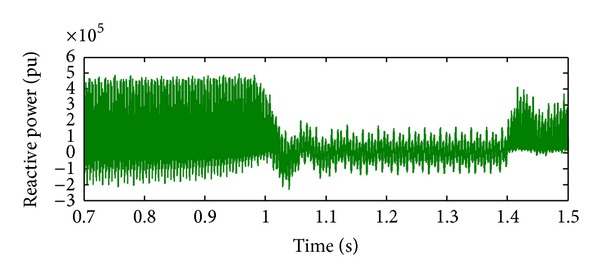
Reactive power during L-G fault.

**Figure 8:**
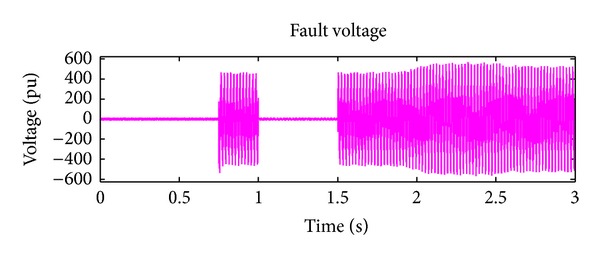
Load voltage during L-G fault.

**Figure 9:**
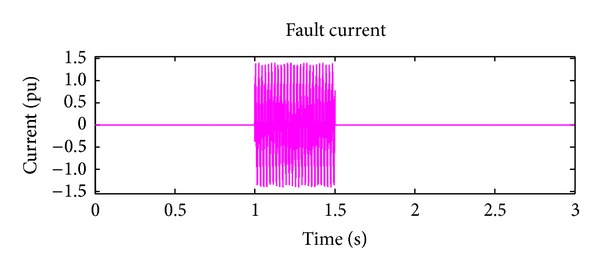
Load current during L-G fault.

*Case 1.* LG fault occurs on phase A. The single line to ground fault occurs on phase A. The duration of the fault period is from 1 sec to 1.5 sec. After 1.5 sec, induction motor load will act, and this results in a gradual increase for a short duration and then it attains constant. The real and reactive power behaviour during faulty period is exposed in Figures 6 and 7. Initially, the voltage on the IM load side is zero as the IM load is included only at 0.75 sec. The IM load voltage is at 440 V as soon as the motor starts but it instantly drops to zero when the L-G fault occurs, that is, at 1 sec. So the load voltage remains at zero until the L-G fault is recovered, that is, till 1.5 sec. After 1.5 sec, the load voltage increases to 440 V. Similarly, during fault event, the fault current will increase to its maximum value of 1.5 mA from 1 sec to 1.5 sec. It will remain zero throughout the operating period as illustrated in Figures 8 and 9.

**Figure 10:**
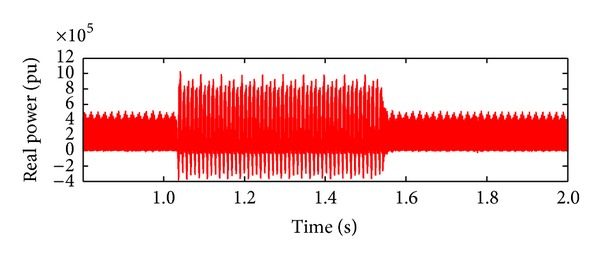
Real power during L-L fault.

**Figure 11:**
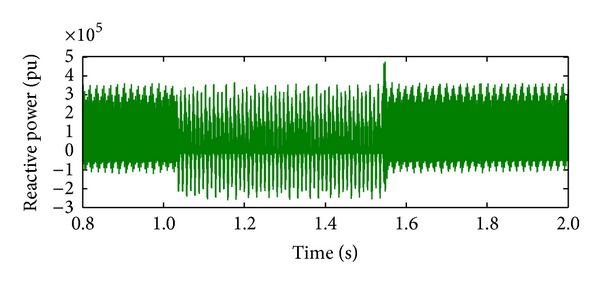
Reactive power during L-L fault.

**Figure 12:**
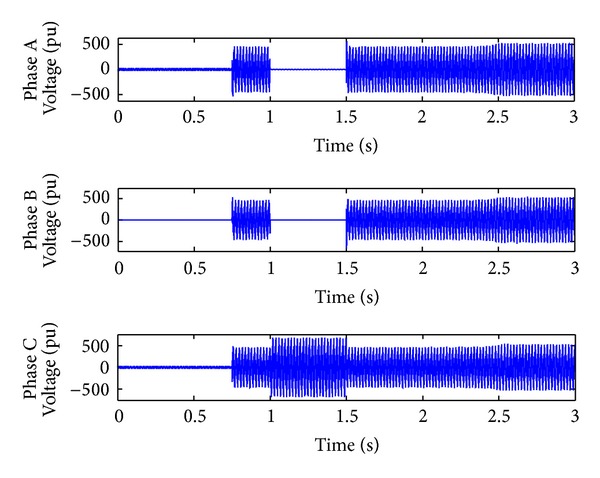
Load voltage during L-L fault.

**Figure 13:**
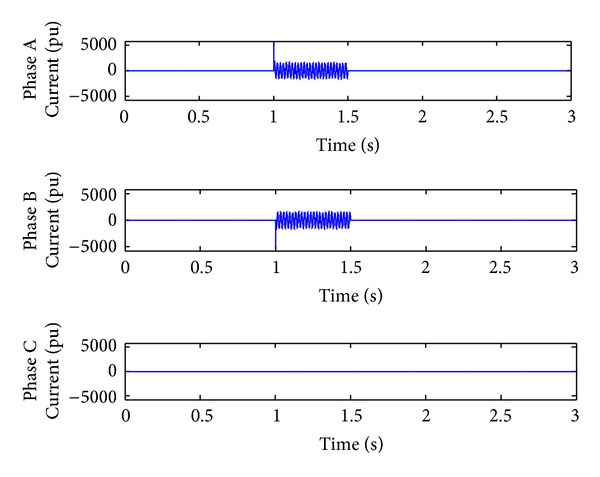
Load current during L-L fault.

*Case 2.* LL fault occurs on phase A and phase B. The line to line fault occurs on phase A and phase B. The duration of the fault period is from 1 sec to 1.5 sec. As illustrated in Figures 10 and 11, initially the real power maintains a constant value till 1 sec. From 1 to 1.5 sec, the real power shoots up drastically about 10 pu. After the fault period, it regains its original value.  Figure 12 illustrates the load voltage on the IM load side performance during fault on phase A and phase B. Voltage of phase A and phase B falls to zero, while voltage swell which exists in phase C can be seen during the faulty condition. In the same way, the current of phase A and phase B shoots up to its maximum value, while the current of phase C remains zero, Figure 13.

**Figure 14:**
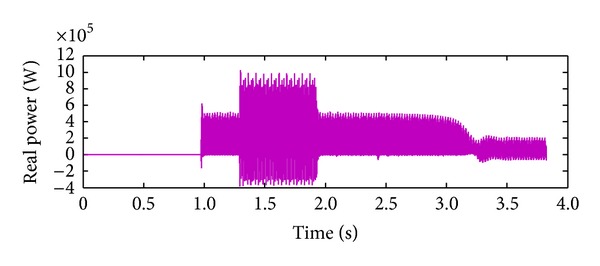
Real power during LLG fault.

**Figure 15:**
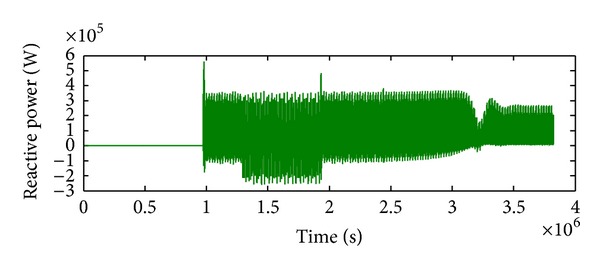
Reactive power during LLG fault.

**Figure 16:**
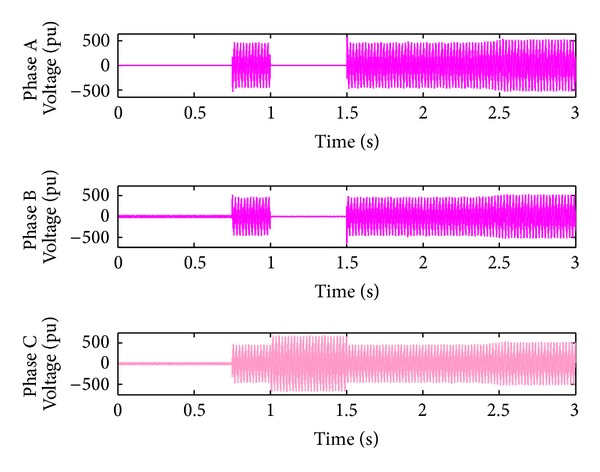
Fault voltage during LLG fault.

**Figure 17:**
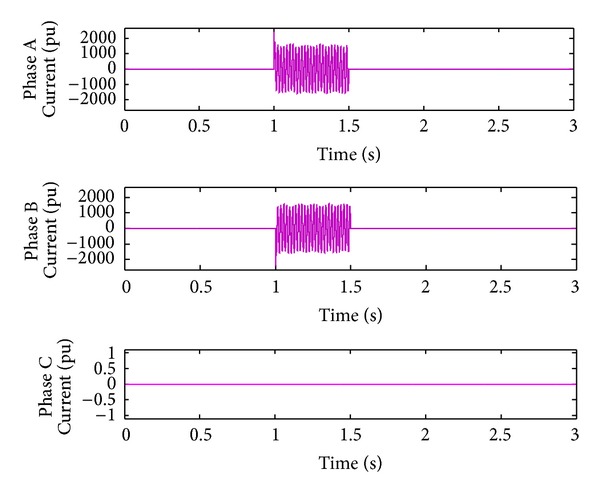
Fault current during LLG fault.

*Case 3.* LLG fault occurs on phase A and phase B to ground. From Figures 14 and 15, the behaviour of the real and reactive power on source side during faulty condition is clearly revealed. Since phase A and phase B were affected by the fault, phase C still continues to supply a maximum permissible power. At 1.5 sec, the circuit breaker connects the system and the IM load is included. As per Figures 16 and 17, the fault voltage and fault current fluctuate accordingly. 

**Figure 18:**
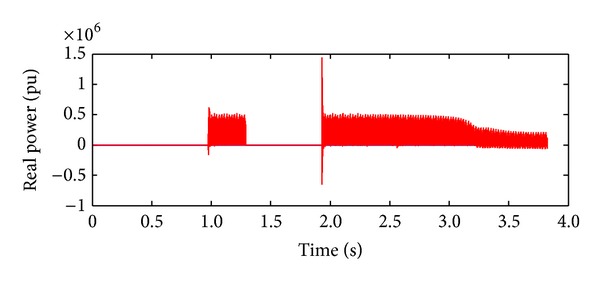
Real power during LLLG fault.

**Figure 19:**
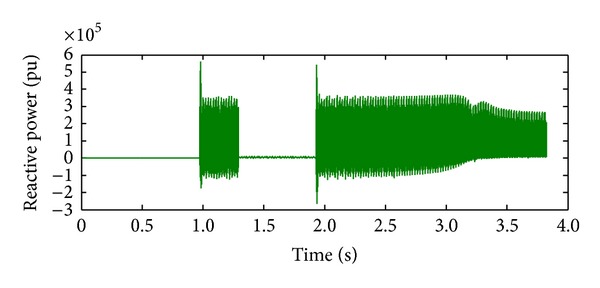
Reactive power during LLLG fault.

**Figure 20:**
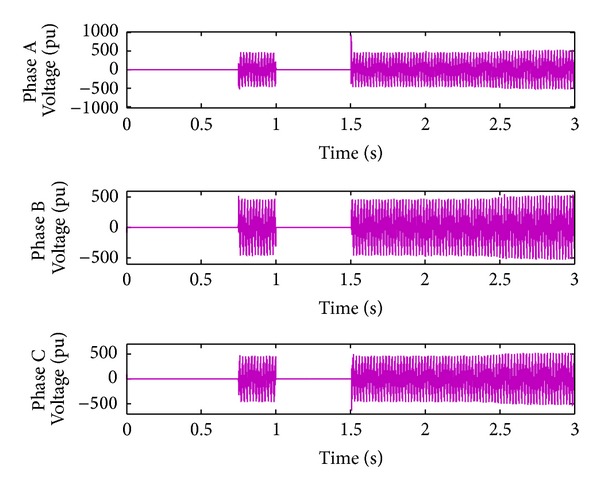
Fault voltage during LLLG fault.

**Figure 21:**
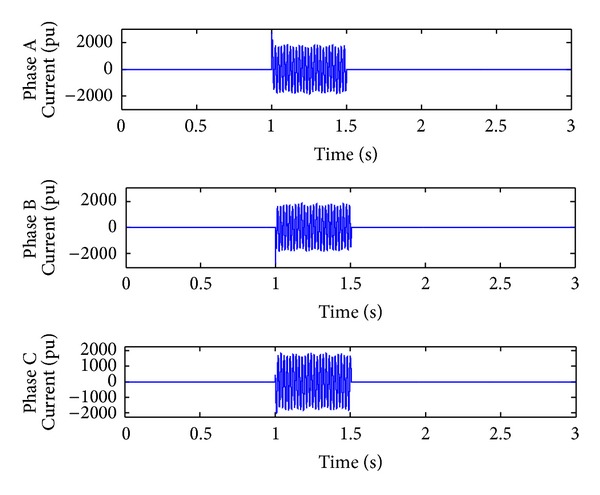
Fault current during LLLG fault.

*Case 4.* LLLG fault—symmetrical fault. For the stability analysis, 3LG fault is also considered along with the asymmetrical fault. The fault occurs at the fault position F1 on the load side as shown in Figure 4. The real and reactive power on the source side and the voltage and current at the time of fault are projected. The real power and reactive power are controlled to the sufficient level during the fault period by means of the proposed droop control strategy. Figures 18 and 19 show the response of real and reactive power. The voltage and current behaviour on the faulty condition are presented in Figures 20 and 21.

## 6. Conclusion

This paper explores the microgrid system behaviour when it is exposed to the consequent faults like symmetrical fault (LLLG) and asymmetrical fault (L-G, LL, and LLG). With this intention to enhance the system performance, droop control technique is adopted. Particularly *P*-*F* droop control technique is enabled for the inverter based DGs. For the simulation results, it is evidence that the droop control strategy enhances the performance of DGs during the various faults conditions and also when the DGs are connected to the IM load and RL load. The simulation results evidenced that the proposed power controller proffers an exceptional response in regulating the microgrid voltage and frequency with an allowable level of harmonic content during autonomous mode.
